# 

**Published:** 1894-11

**Authors:** 


### CONTENTS.

COMMUNICATIONS.

Syphilis ........ 521
Local Anæsthetics ........ 527
Porcelain Work ........ 532

PROCEEDINGS.

Southern Dental Association—1894 ........ 538
The American Dental Association ........ 550
The National Association of Dental Examiners ........ 564

NOTICES.

American Dental Society of Europe ........ 569
Dr. J. Austin Dunn ........ 537

EDITORIAL.

Union ........ 569
Ohio State Dental Society ........ 570
Dental Colleges—Opening ........ 571

